# A Transcriptome Survey Spanning Life Stages and Sexes of the Harlequin Bug, *Murgantia histrionica*

**DOI:** 10.3390/insects8020055

**Published:** 2017-05-25

**Authors:** Michael E. Sparks, Joshua H. Rhoades, David R. Nelson, Daniel Kuhar, Jason Lancaster, Bryan Lehner, Dorothea Tholl, Donald C. Weber, Dawn E. Gundersen-Rindal

**Affiliations:** 1 Invasive Insect Biocontrol and Behavior Laboratory, USDA-ARS, Beltsville, MD 20705, USA; michael.sparks@ars.usda.gov (M.E.S.); joshua.rhoades@ars.usda.gov (J.H.R.); daniel.kuhar@ars.usda.gov (D.K.); don.weber@ars.usda.gov (D.C.W.); 2 Department of Microbiology, Immunology and Biochemistry, University of Tennessee Health Science Center, Memphis, TN 38163, USA; drnelson1@gmail.com; 3 Department of Biological Sciences, Virginia Polytechnic Institute and State University, Blacksburg, VA 24061, USA; jlancas7@vt.edu (J.L.); lehnerb@vt.edu (B.L.); tholl@vt.edu (D.T.)

**Keywords:** harlequin bug, invasive insects, agricultural pest, RNAi targets, insect pheromones, xenobiotic detoxification, transcriptomics

## Abstract

The harlequin bug, *Murgantia histrionica* (Hahn), is an agricultural pest in the continental United States, particularly in southern states. Reliable gene sequence data are especially useful to the development of species-specific, environmentally friendly molecular biopesticides and effective biolures for this insect. Here, mRNAs were sampled from whole insects at the 2nd and 4th nymphal instars, as well as sexed adults, and sequenced using Illumina RNA-Seq technology. A global assembly of these data identified 72,540 putative unique transcripts bearing high levels of similarity to transcripts identified in other taxa, with over 99% of conserved single-copy orthologs among insects being detected. Gene ontology and protein family analyses were conducted to explore the functional potential of the harlequin bug’s gene repertoire, and phylogenetic analyses were conducted on gene families germane to xenobiotic detoxification, including glutathione S-transferases, carboxylesterases and cytochrome P450s. Genic content in harlequin bug was compared with that of the closely related invasive pest, the brown marmorated stink bug, *Halyomorpha halys* (Stål). Quantitative analyses of harlequin bug gene expression levels, experimentally validated using quantitative real-time PCR, identified genes differentially expressed between life stages and/or sexes.

## 1. Introduction

The harlequin bug, *Murgantia histrionica* (Hahn) (Hemiptera: Pentatomidae), though native to Mexico and Central America, has been a serious pest of mustard-family crops (Brassicaceae) in the southern USA for ~150 years, and also infests plants in the caper family (Capparaceae) [1,2,3]. It is limited in its northern spread by cold winter temperatures, and through the mid-20th century, there were destructive harlequin bug outbreaks following mild winters [3,4,5,6]. Earlier still, the insect’s unexpected arrival in Confederate states during the Civil War prompted a formal accusation that the Union had introduced it as a deliberate act of biological warfare [7].

Destruction of crop debris in which bugs overwinter, use of soaps and oil emulsions, handpicking, and trap cropping with attractive plants such as early cruciferous crops, were recommended controls in the 19th and early 20th centuries [3]. With the advent of synthetic organic insecticides, harlequin bug was suppressed by broad-spectrum organophosphates and carbamates, and later by pyrethroids and neonicotinoids [3]. However, its recently increasing pest status reflects its tolerance of more selective management tactics for other pests of cole crops, chiefly aphids and caterpillars, and the prevalence of organic vegetable culture. Natural enemies are limited to hymenopterous egg parasitoids and generalist predators [8]. Harlequin bugs sequester glucosinolates from their host plants [9], accounting for their red-and-black aposematic coloration and possibly for the lack of nymphal or adult parasitoids known to attack other pentatomids.

With continued insecticide usage, rising populations under selective management tactics, and limited suppression by natural enemies, the need to develop alternative control mechanisms for this insect pest is becoming increasingly urgent. The development of effective, species-specific and environmentally friendly molecular biopesticides offers a possible alternative (or complement) to chemical pesticides. RNA interference (RNAi) involves the targeting of specific host genes using double-stranded RNA, thereby effecting temporary knockdowns of gene expression and associated gene function [10]. Prerequisite to identifying specific gene targets is the availability of high quality, annotated gene sequence and expression data, which for harlequin bug has heretofore been unavailable.

Pheromone biosynthesis-related genes constitute one such family of potential gene targets. In a laboratory setting, Zahn et al. [11,12] showed that an aggregation pheromone emitted by adult males feeding on a plant host was attractive to both sexes of adults, and concluded that the pheromone was a single isomer of 10,11-epoxy-1-bisabolen-3-ol [4-[3-(3,3-dimethyloxiran-2-yl)-1-methylpropyl]-1-methylcyclohex-2-en-1-ol], dubbed murgantiol. Khrimian et al. [13] showed that the pheromone consisted of two stereoisomers, (3*S*,6*S*,7*R*,10*S*)- and (3*S*,6*S*,7*R*,10*R*)-10,11-epoxy-1-bisabolen-3-ol, which occur in a ~1.4:1 ratio in emissions of adult male harlequin bug. Weber et al. [14] demonstrated that, in the field, lures containing a synthetic eight-stereoisomer blend were of comparable attractiveness with pure pheromone components to both sexes in adults, as well as to nymphs, and that presence of a host plant (collard) increased this attractiveness. Cabrera Walsh et al. [15] found that, although harlequin bugs were strongly attracted to host plants by their pheromone lure, they were not retained more on plants with pheromone than those without it, an important consideration for trap cropping and pesticide application strategies.

**Figure 1:**
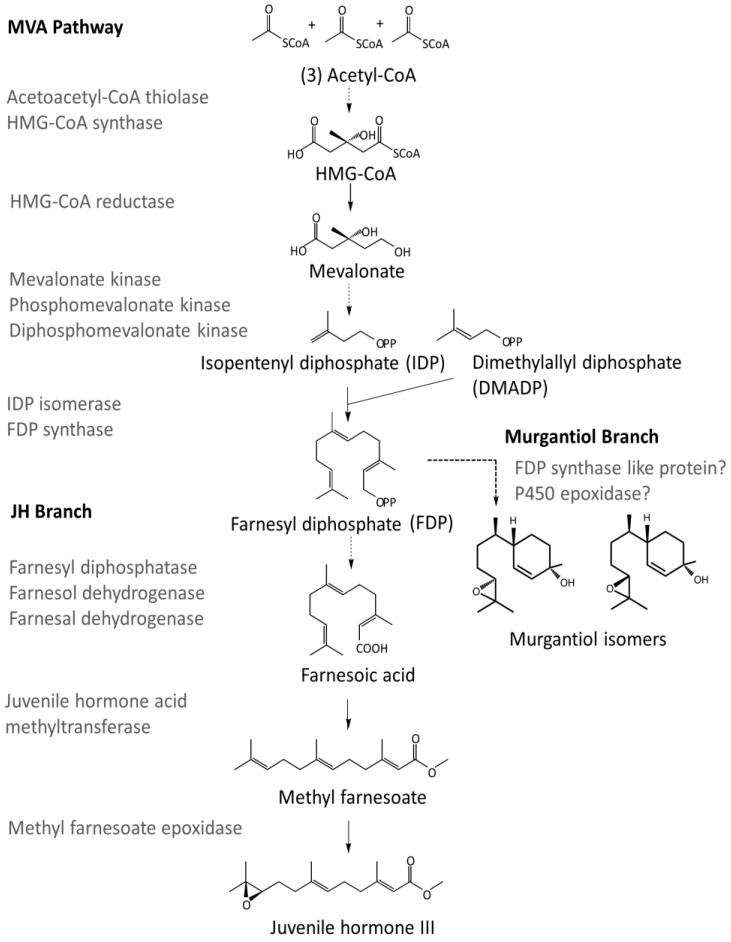
Mevalonate (MVA) and Juvenile Hormone (JH) biosynthetic steps and the proposed pathway for the formation of the aggregation pheromone murgantiol. Multiple enzyme conversions are represented with dashed arrows.

As an epoxybisabolenol, murgantiol is most likely synthesized via the mevalonic acid (MVA) pathway as the core terpenoid biosynthetic pathway in insects [16] (Figure 1). The six enzymatic steps of this pathway convert acetyl-CoA to the universal 5-carbon terpenoid building block, isopentenyl diphosphate (IDP). Enzymatic condensation of two units of IDP with one unit of its isomer dimethylallyl diphosphate (DMADP) gives rise to farnesyl diphosphate (FDP), which is the central 15-carbon precursor in juvenile hormone (JH) biosynthesis (Figure 1). Based on recent findings by Beran et al. [17] on the biosynthesis of sesquiterpene aggregation pheromones in flea beetle, it can be assumed that FDP is also a precursor of murgantiol in a pathway that includes steps catalyzed by isopentenyl diphosphate synthase (IDS) type and cytochrome P450s (CYP) epoxidase enzymes (Figure 1).

Another class of gene targets involves enzymes used in detoxification of xenobiotic compounds, which includes synthetic insecticides. Glutathione S-transferases (GSTs), carboxylesterases (COEs), and CYPs are three classes of enzymes that have been widely associated with rising levels of insecticide resistance, and their characterization is of urgent concern in maintaining feasible and effective pest management programs. In particular, RNAi-mediated knockdown of these genes may result in more efficacious and/or efficient use of chemical pesticides, thereby reducing the monetary and environmental costs associated with their use in modern agricultural production operations.

In insects, GSTs are typically split into two groups: cytosolic and microsomal. Cytosolic GSTs are ordinarily divided into six subclasses: Delta, Epsilon, Sigma, Omega, Theta and Zeta [18,19]. In particular, Delta and Epsilon groups are unique to insects and have been widely associated with insecticide resistance; although the Delta type are observed among the Insecta in general, the Epsilon subclass appears to be unique to the Holometabola [18]. In contrast to cytosolic GSTs, those of the microsomal type are membrane-associated, and, although they are functionally similar to cytosolic GSTs, they are not currently implicated in insecticide resistance.

Insecticide resistance among hemipterans has been associated with multiple carboxylesterase groupings. Point mutations of acetylcholinesterases, as well as gene duplication and over-expression of E4 and FE4 β-esterases, have been reported in *Myzus persicae* [20] and *Aphis gossypii* [21]. The most recent classification of carboxylesterases divides these genes into three major clades [22,23,24]. The first and most basal is the neurodevelopment and cell adhesion clade, which comprises neuroligins, glioactins, neurotactins, acetylcholinesterases and glutacin enzymes. With the exception of acetylcholinesterases, all members of the neurodevelopmental clade are non-catalytic. The second is the hormone and semiochemical processing clade, which contains secreted β-esterases, integument esterases and juvenile hormone esterases. The third clade is associated with dietary and detoxification functions and contains α-esterases.

Cytochrome P450s have a two-fold role in gene-environment interactions, participating both in host biosynthetic pathways, as well as in detoxification of xenobiotic compounds. Biosynthesis of critical endogenous molecules via P450s can be targeted by pesticides. Molting, hormone/pheromone synthesis and turnover, and cuticular hydrocarbon waterproofing may all be targets to exploit for pest control. Conversely, insects have evolved modified P450s to detoxify exogenous chemicals like pesticides, leading to resistance. These two classes of P450s are easily observed in phylogenetic trees. Highly conserved one-to-one orthologs between insect species are parts of pathways to make essential biomolecules like ecdysone (Halloween genes: CYP302, CYP306, CYP307, CYP314, and CYP315; [25]), juvenile hormone (CYP15; [26]), as well as fatty-acid-derived alkanes and alkenes for exoskeleton coating (CYP4G; [27,28]).

Resistance has been associated with numerous cytochrome P450s, often members of “gene blooms”, which are large expansions of P450s in tandem duplication arrays on chromosomes. These are not highly conserved or even limited to one CYP clan. Almost any P450 family can become adapted to detoxify a pesticide [29,30,31]. Resistance may not only be due to pesticide inactivation, but it may be caused by blocking pesticide entry via thickening of the cuticular hydrocarbon barrier [32]. On the biocontrol side, entomopathogenic fungi kill insects by using P450s like CYP52X1 to degrade and penetrate the hydrocarbon coating on insects [33].

Mating in many if not most insects is dependent on pheromone signaling. The volatile chemicals must be synthesized, released and rapidly turned over at the receptors in antennae. P450s are actively involved in both areas [34,35,36]. Even the status of the queen in social insects, such as the termite *Cryptotermes secundus*, depends on molecules made by P450s [37]. Disruption of these signals by P450 inhibitors may also constitute an effective pesticidal mode of action.

In this study, we provide the first large scale assembly of transcriptomic sequences in the important agricultural pest, *Murgantia histrionica* (Hahn). We also identify a number of pheromone synthesis- and insecticide resistance-related genes with potential utility in the development of molecular biopesticides and/or transgenic organisms for insect biocontrol.

## 2. Materials and Methods

Field-collected harlequin bugs were cultured for several generations in a laboratory setting without exposure to insecticides. Original collections were by hand from their host plants in gardens and small farms within 80 km of Beltsville, MD. They were reared under 16:8 L:D photoperiod on collards in a greenhouse with temperature 18 to 28 °C. Messenger RNA populations were extracted from whole-insect preparations from 2nd and 4th instar nymphs, as well as female and male adults. For 2nd instar nymphs, 15 insects were pooled per bioreplicate, whereas five insects were pooled for all other samples. Only one bioreplicate was submitted for PE100 transcriptome sequencing (i.e., paired-end reads, 100 bp apiece) on an Illumina HiSeq instrument: RNA libraries were prepared using the TruSeq RNA v2 kit per manufacturer’s protocol (Illumina, San Diego, CA, USA). Total RNA quality was checked using an Agilent Bioanalyzer 2100 (Agilent, Santa Clara, CA, USA), and enrichment for mRNA was done using poly-dT beads. Libraries were resuspended in Qiagen EB buffer (Qiagen, Germantown, MD, USA) and a Fragment Analyzer NGS (Advanced Analytical, Ankeny, IA, USA) was used to check library size and quality; library concentration was checked using a Qubit fluorometer (ThermoFisher Scientific/Life Technologies, Waltham, MA, USA).

Raw sequencing volumes achieved are shown in Table 1, and these sequencing data were uploaded to the NCBI Sequence Read Archive (SRA) division under the BioProject accession identifier, PRJNA302154. Raw reads were normalized in a sample-specific manner using utilities supplied with the Trinity RNA-Seq assembly software package, version 2.2.0 [38]. A global transcriptome assembly was produced using Trinity: default parameter settings were used, with the exceptions that the read normalization (as here applied to the set of pooled reads) and Trimmomatic-based quality control submodules were invoked as pre-processing steps [39]. The resulting putative unique transcripts (PUTs) were post-processed using PRINSEQ-lite [40], which in particular was used to clip any residual poly-A/T tails. Resultant PUTs having lengths of at least 200 bp were then compared with the UniVec database using the NCBI C++ Toolkit’s vecscreen utility [41]—transcripts having hits flagged as “Strong” and/or “Moderate” were purged. Residual transcripts were checked for contaminants and low-quality elements using the NCBI Transcriptome Shotgun Assembly (TSA) division’s internal screening system, and assessed for completeness with respect to known single-copy orthologs generally conserved among insects using the BUSCO pipeline [42]. The final assembly was also made publicly available under the aforementioned BioProject accession.

NCBI-vetted PUTs were compared with the NR protein database using Blastx [43] with default parameter settings. PUTs were partitioned into gold, silver and bronze tiers accordingly to similarity with existing sequence data as described previously [44,45]: Briefly, each gold-tier entry involved a PUT at least 300 nucleotide residues long exhibiting a single high-scoring segment pair in its Blastx hit against a reference protein of at least 100 amino acids; at least 75% of aligned residues had to be positively similar, with the ratio of hit length to subject sequence length being at least 90%. Silver-tier PUTs were a minimum of 100 nucleotide residues long with a hit spanning at least 75% of an NR reference protein sequence’s length. Bronze-tier entries involved PUTs at least 100 nucleotides long with hits covering at least 30% of the NR protein’s length.

Protein family analyses were conducted using HMMER3 with default settings in conjunction with the Pfam-A (version 30) database [46,47]. Each PUT could be associated with zero, one or more distinct Pfams. Inferred translational products identified in the gold-tier PUT subset were analyzed for Pfam family content. An additional analysis was performed using the complete set of PUT sequences by comparing the longest ORF present in each (considered across all six frames) with Pfam-A. Annotations based on gene ontology (GO) terms were determined using the pfam2go mapping table made available by Pfam, for each of the ontology’s cellular component, biological process and molecular function aspects. Fine-grained GO terms were resolved to their respective penultimate ancestral nodes in the directed acyclic graph (DAG) used to represent the ontology, thereby providing a more abstract view of the functional capacity inherent in the harlequin bug transcriptome—more specifically, these GO-Slim terms were recovered through traversals of “is_a” paths in the DAG using custom scripts.

Isoform-level expression amounts were estimated using the RSEM package and were recorded using the Transcripts per Million (TPM) measure [48]. Three contrasts were made based on these abundance estimates: 2nd vs. 4th instar nymphs, female vs. male adults, and nymphs vs. adults—reads from 2nd and 4th instar nymphs were pooled to compose the “nymphal” group, and those from adult males and females for the “adults” set. A listing of all PUTs exhibiting at least a two-fold expression differential is presented in Table S1. Validation of gene expression differences was performed on a subset of 16 transcripts using quantitative real-time PCR, incorporating three biological replicates and three technical replicates. Primers were designed using PrimerPlex version 2.62 (PREMIER Biosoft, Palo Alto, CA, USA); these data are also presented in Table S1. Following manufacturer protocol, cDNA was synthesized using SuperScript^TM^ II Reverse Transcriptase (ThermoFisher Scientific). Primers targeting harlequin bug 18S rRNA (forward: 5′-TTTTATCCAGAAAATCCCGATCA-3′, reverse: 5′-ACAACAAGTCCTCCGAAAAACC-3′) were used to control for RNA quantity, and those targeting harlequin bug elongation factor 1-alpha (forward: 5′-CGAGAAAGAGGCTCAGGAGA-3′, reverse: 5′-TCAGCCTGAGAAGTCCCTGT-3′) were used as an internal reference for gene expression. Real-time PCR was ran on an ABI 7500 instrument using Power SYBR Green master mix (ThermoFisher Scientific), as follows: 40 cycles of 50 °C for 2 min, 95 °C for 10 min, 95 °C for 15 s, 60 °C for 1 min; followed by 95 °C for 15 s, 60 °C for 1 min, 95 °C for 30 s, and finally 60 °C for 15 s.

To identify harlequin bug transcripts involved in insecticide resistance, proteins identified in the Gnomon-based annotation of the brown marmorated stink bug (*Halyomorpha halys*) genome as being carboxylesterases (COE) or glutathione-S-transferases (GST) were retrieved. Blastp was used to search the full *H. halys* inferred protein set to identify any COE or GST proteins that had not been properly labeled as such by automated methods. Following manual scrutiny, these refined sets were used as Blastp queries against the harlequin bug data—in particular, *H. halys* sequences were compared against the longest ORF present in each harlequin bug transcript to test for homology. Inclusion of an *M. histrionica* gene in the COE or GST gene family required its top high-scoring segment pair to exhibit a bit score of at least 75, a subject sequence length of at least 100 amino acid residues, a hit length to subject sequence length ratio of 0.90 or greater, a hit length to query sequence length ratio of 0.75 or greater, and a ratio of positively similar aligned residues to hit length of at least 0.80. Independently for each of these gene families, combined *M. histrionica* and *H. halys* protein sets were multiply aligned using the MUSCLE program [49], from which a maximum likelihood-based phylogeny was generated using the method of Le and Gascuel [50] as implemented in PhyML [51] using 100 bootstrap replicates. Phylogenies were visually rendered using the R package, phytools [52]. For both gene family analyses, GenomeThreader [53] was used to align *M. histrionica* proteins to the brown marmorated stink bug genome (available at https://i5k.nal.usda.gov/Halyomorpha_halys) in an effort to elucidate patterns of molecular evolution across this taxonomic divide.

The full PUT set was batch tBlastn searched with each cytochrome P450 subfamily from *Halyomorpha halys* (brown marmorated stink bug; 48 sequences). The top 50 hits for each search were kept using an expect value of 10. Blast results were imported into a spreadsheet and duplicate accessions were removed by filtering for unique entries only. In total, 478 PUTs were identified. These sequences were batch Blastx searched against 9125 named insect P450 sequences and the best hit was retained with percent identity and alignment length. The results were sorted by best blast hit which clustered closely related sequences together. The longest member of each group was translated and used to search the 478 PUTs for exact or near-exact matches. These sequences were binned into a collection from a single gene. Additional P450 PUTs were identified by HMMER searches of Pfam-A and Blastx searches of the NCBI protein database, NR. These were mostly short (i.e., <175 aa) fragments.

To determine whether harlequin bug harbors an iflavirus similar to that observed in the brown marmorated stink bug [54], raw RNA-Seq reads were mapped against the viral genome sequence available under NCBI Accession number KF699344.1 using bowtie2 [55]. PUTs potentially associated with organisms of the genera *Pantoea*, *Erwinia* and *Nosema*, which correspond to known pentatomid symbionts [45,56,57], were identified through Blastx comparisons with representative sequences present in the NR database. These comparisons involved the full set of assembled PUTs rather than those that had been explicitly vetted by the NCBI TSA, a vetting which involves removal of putative contaminant sequences, comprising microbial symbionts.

## 3. Results

Assembly of the full RNA-Seq dataset generated in this study produced 526,403 putative unique transcripts (PUTs) comprising 427,532,460 bases. Following data cleaning routines, a total of 523,102 NCBI-vetted PUTs, containing 425,689,726 assembled bases, was made available under BioProject accession number PRJNA302154. A total of 95.7% of conserved single-copy orthologs in the Insecta class were fully represented in this assembly, with 3.6% fragmented and only 0.7% absent. Sorting these sequences on the basis of extrinsic homology information resulted in the labeling of 19,303 gold-tier, 19,755 silver-tier and 33,482 bronze-tier PUTs. Pfam family analysis of translation products inferred from gold-tier PUT sequences identified 4518 unique families in total. Assaying Pfam family presence among longest ORFs present in the comprehensive PUT collection resulted in detection of 5352 distinct Pfam families in total. Table 2 presents the 25 most abundant Pfam entries detected in both datasets. These described a fairly typical array of protein family content, evincing presence of genes associated with cell signaling, DNA binding and innate insect immunity.

**Figure 2:**
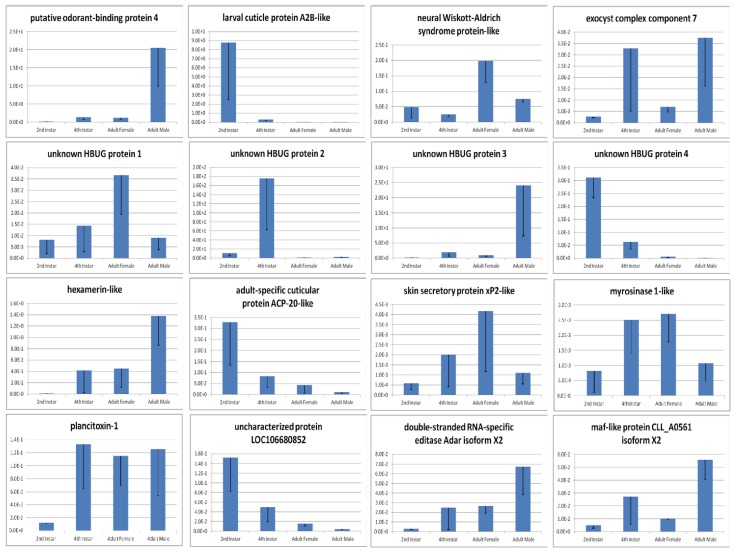
Quantitative real-time PCR validation of select harlequin bug transcript expression patterns.

Analysis of the complete PUT dataset yielded 552 distinct GO terms associated with the ontology’s Biological Process aspect, 199 with Cellular Component and 627 with Molecular Function. Analysis on the gold-tier PUT subset resulted in identification of 463, 162 and 554 distinct terms corresponding to the Biological Process, Cellular Component and Molecular Function aspects, respectively. Supplementary Figure S1 presents the 10 most frequently encountered GO terms seen within each of these dimensions, for each set of PUTs; no appreciable differences were evident with which to distinguish functional capacity of the complete PUT set relative to the gold-tier subset. Analogous to its full-ontology counterpart, Supplementary Figure S2 presents the five most abundant GO-Slim terms observed. Similarly, the distribution of GO-Slim terms between the two datasets appeared equitable. Of the PUTs that exhibited the greatest fold changes in expression levels between 2nd and 4th instar nymphs, the transcripts seemingly most abundantly up-regulated in the former relative to the latter comprised a cuticular protein, perhaps associated with the molting process (see Table 3). Those seemingly more abundantly expressed among 4th instar nymphs were usually digestion or stress-response related. Transcripts predominantly expressed in females included a juvenile hormone acid O-methyltransferase and a muscle component, troponin C. Transcripts that suggested male-dominant expression patterns included an odorant binding protein, which may be involved in mate recognition. No clear functional trend seemed evident among genes whose fold changes suggested more abundant expression in adults relative to nymphs, but in the reverse case, transcripts were often associated with cuticular tissues. In all three comparisons, transcripts with the greatest perturbations in expression fold changes included a number of distinct uncharacterized proteins. Gene expression patterns for a subset of these genes were validated experimentally using quantitative real-time PCR, and these results generally corroborated findings suggested by RNA-Seq data (see Figure 2).

**Figure 3:**
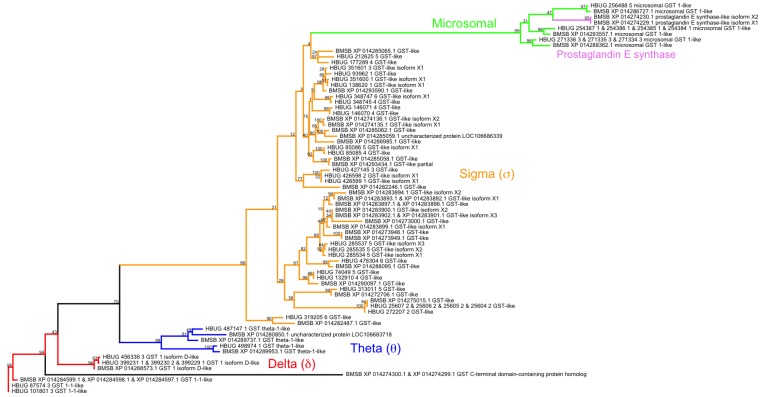
Maximum likelihood-based glutathione S-transferase phylogeny for *H. halys* and *M. histrionica* proteins. Four distinct clades corresponding to GST class were observed: delta (red), theta (blue), sigma (brown) and microsomal (green). Two prostaglandin E synthase isoforms (pink) were placed in the microsomal GST clade. Bootstrap support (100 replicates) is indicated on branches.

Forty-one *H. halys* and 44 *M. histrionica* glutathione S-transferase associated transcripts encode 35 and 34 unique protein sequences, respectively. A phylogeny of these genes’ unique translation products is shown in Figure 3. Four distinct clades corresponding to GST class were observed: Theta, Delta/Epsilon, Sigma, and microsomal (which was positioned within the Sigma clade). Although members of the Delta/Epsilon classes are not distinguished in annotations made available by the NCBI, the observation that Epsilon-type GSTs are exclusive to the Holometabola [18] suggests genes from these two Paraneopteran species are of the Delta type. The majority of protein sequences were placed in the Sigma clade. Two *H. halys* prostaglandin E synthase isoforms were observed and subsumed under the phylogeny’s microsomal GST glade; no harlequin bug homologs to these enzymes were evident, however. Thirty-one of the 44 unique *M. histrionica* GST associated protein sequences could be reliably splice-aligned to the *H. halys* genome, but only 19 of these aligned to unique loci. After further accounting for isoforms, an additional 14 GST homologs were identified: one Theta, two Delta, eight Sigma and three microsomal GSTs. Homologs of GSTs from the Zeta and Omega subclasses were not apparent in these data.

Ninety *H. halys* and 88 *M. histrionica* carboxylesterase associated transcripts encode 82 and 70 unique translation sequences, respectively. A maximum likelihood phylogeny of these unique translation products is shown in Supplementary Figure S3. A preponderance of sequences were phylogenetically positioned in a clade containing carboxylesterases proper, comprising carboxylesterase 4A-like, carboxylesterase 5A-like and β-esterase clades. The large β-esterase clade contains venom carboxylesterase 6-like enzymes, as well as E4 and FE4 esterases. Acetylcholinesterase-, neurotactin- and neuroligin-associated sequences were all basal to the large carboxylesterase clade.

Fifty-five of the 70 unique *M. histrionica* carboxylesterase associated transcripts map to the *H. halys* genome, suggesting two acetylcholinesterase (four mapped transcripts), three neuroligin (11 mapped transcripts), one neurotactin (three mapped transcripts) and 19 carboxylesterase (37 mapped transcripts) homologs. All fifteen unique, unmapped *M. histrionica* carboxylesterase associated transcripts are located within the carboxylesterase clade, perhaps suggesting a lineage-specific expansion of these genes in harlequin bug (see Supplementary Figure S3).

**Figure 4:**
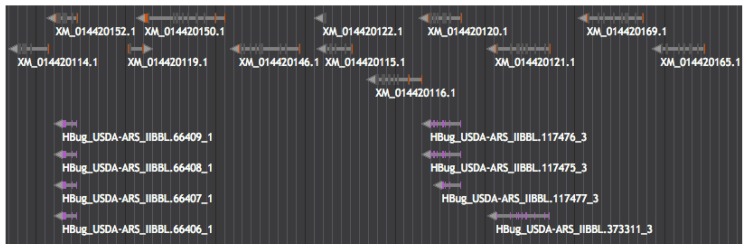
Segment of scaffold 261 from the *Halyomorpha halys* genome assembly harboring an array of eleven E4/FE4 esterase genes. The *H. halys* COE genes are show in the top track, in grey (note that gene model XM_014420119.1 appears to correspond to an unrelated lipase gene) a total of six unique *M. histrionica* proteins (bottom track, purple) could be aligned to this chromosomal region, and these associated with three distinct COE loci present in the *H. halys* genome sequence.

Some *H. halys* scaffolds contain multiple esterase genes in close proximity, specifically scaffolds 261 and 2592, which comprise tandem arrays of eleven and six esterase genes, respectively. On both scaffolds 261 and 2592, *M. histrionica* esterase proteins mapped to three distinct, homologous esterase *H. halys* loci, suggesting a potentially elevated esterase gene copy number in this latter species relative to the former (see Figure 4).

*H. halys* scaffolds 261, 2592, 159, 1429 and 116 contain, respectively, 11, 6, 6, 4 and 2 carboxylesterase genes in close proximity. On both scaffolds 261 and 2592, *M. histrionica* carboxylesterase associated transcripts map to three distinct, homologous *H. halys* carboxylesterase associated loci (see Figure 4). All other *M. histrionica* carboxylesterase associated transcripts map to *H. halys* scaffolds containing just one carboxylesterase associated loci.

**Figure 5:**
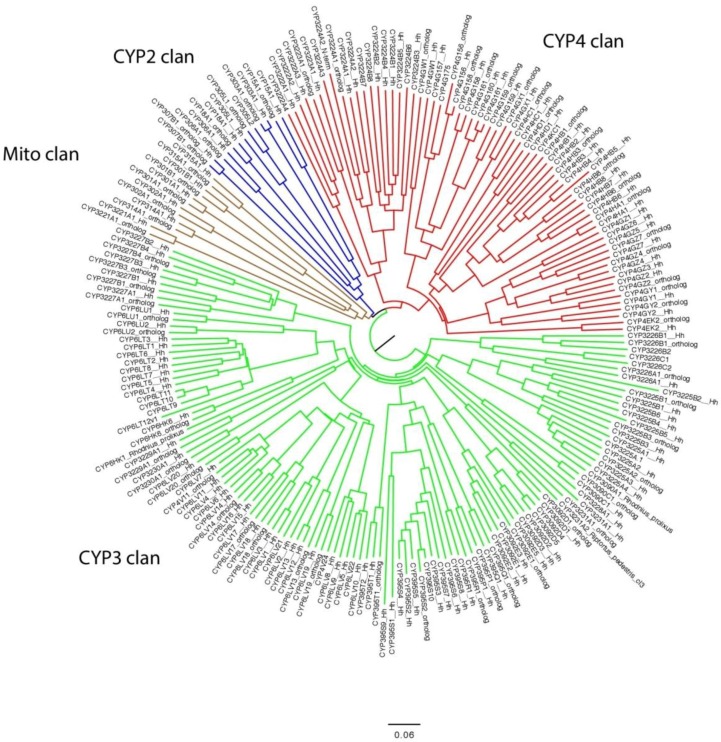
Cytochrome P450s from *H. halys* (126 sequences) and *M. histrionica* (86 sequences) plus two from *Rhodnius prolixus* and one from *Riptortus pedestris* were aligned using Clustal Omega (http://www.ebi.ac.uk/Tools/msa/clustalo/) at EBI. An NJ midpoint-rooted tree was made. The tree was drawn using Figtree v1.3.1 and labeled in Adobe Illustrator CC ver17.0.0. P450 clans are colored. Sequences less than 175 amino acids were not included.

A total of 133 novel cytochrome P450 sequences in *M. histrionica* were assigned names based on their relationship to named P450s from other insects. Many were orthologs to P450s of *Halyomorpha halys.* Every P450 family in brown marmorated stink bug was found in harlequin bug except CYP3228A1. This sequence was searched for specifically and found as a pseudogene fragment. An NJ tree was made using Clustal Omega using midpoint rooting (Figure 5). The four insect clans are present. The CYP2 clan and the mito clans are small, containing all of the Halloween genes and some other highly conserved genes like CYP15, CYP18, CYP301 and CYP305. The CYP3 clan is large containing about half of the sequences with a gene bloom in the CYP6LV subfamily. The CYP4 clan has about one quarter of the total sequences. These two clans contain the least conserved genes with the exception of the CYP4G genes (cuticular hydrocarbon synthesis).

A number of P450-encoding transcripts exhibited possible sex-preferential gene expression patterns. Five exhibited gene expression patterns suggestive of female-specific expression among adult insects, and seven suggested a male-dominant pattern (see Table 4). Table 4 also presents the seven PUTs having expression values of at least 5 TPM in both male and female imagoes, and exhibited at least a two-fold expression differential per RNA-Seq data—among these, six (~86%) suggested a male-dominant pattern.

In searching for *M. histrionica* terpene biosynthetic genes, a total of 19 sequences were identified using query sequences for the MVA and JH biosynthetic pathways from various insects (Table 5). These sequences were orthologs to respective genes in *H. halys* and included transcripts of all six genes of the MVA pathway (acetoacetyl-CoA thiolase, HMG-CoA synthase, HMG-CoA reductase, mevalonate kinase, phosphomevalonate kinase and diphosphomevalonate decarboxylase) and an IDP isomerase. Furthermore, two IDS homologs of FDP synthase-like genes were observed, one of which exhibited higher expression levels in adults and male bugs (Table 6). In the JH pathway branch, multiple isoforms of farnesol dehydrogenase (3), farnesal dehydrogenase (2), and farnesoic acid methyltransferase (3) were found, but expression values for several of these isoforms were low at all stages of development (Table 6). A single transcript for methyl farnesoate epoxidase was identified; however, no homolog of farnesyl diphosphatase could be observed. While transcripts of MVA pathway genes did not show major developmental or sex specific expression differences, more distinct expression patterns were found for isoforms of JH biosynthetic genes (farnesol dehydrogenase, farnesal dehydrogenase, farnesoic acid methyltransferases) dependent on instar, nymphal/adult stage or sex. As in *M. histrionica*, MVA pathway gene orthologs in *H. halys* were equally expressed among sexes and developmental stages, while expression profiles of orthologs in JH biosynthesis differed dependent on the isoform. These expression patterns were, however, not consistent with those found in harlequin bug. Interestingly, a total of four FDP synthase-like transcripts were detected in *H. halys* as opposed to only two such sequences in *M. histrionica*.

Transcripts appearing to encode proteins involved in RNAi-related processes were detected, including such RISC-associated components as Dicer, Loquacious and Argonaute, as well as homologs of Aubergine, Tarbp2, various RNA helicases and PIWI-containing proteins. No PUTs with evident homology to *sid-1* or *sid-2* were detected, nor were any apparent RNA-dependent RNA polymerases observed.

No evidence of an iflavirus homologous to that previously observed in the brown marmorated stink bug was apparent in harlequin bug, and only very weak evidence supporting the possible presence of a *Nosema* sp. microbe was present: only seven PUTs corresponded to genes observed in the *Nosema* genus, and these were predominantly associated with retrotransposable elements. In contrast, 653 and 67 PUTs exhibited high levels of sequence similarity with known *Pantoea* and *Erwinia* genes, respectively, consistent with the notion that these genera likely comprise the primary microbial endosymbionts of *M. histrionica*, as well as several other pentatomids [57,58].

## 4. Discussion

The increasing importance of stink bug pests is concurrent with the reduction in foliar insecticides in major, predominantly transgenic field crops, and the invasion of several major stink bug pests into new continents. Panizzi et al. [59] listed 25 “important” pest species in 17 genera, and about 41 “less important” pests in 16 additional genera, infesting a wide variety of agronomic and horticultural crops and other useful plants. Since that review, two of those pests hitherto considered less important, *H. halys* and *Bagrada hilaris*, have become vastly more important following continental-scale invasions, and a number of other species are potentially serious threats for invasion [59,60]. Complicating management is the fact that several predatory pentatomid species in the subfamily Asopinae are important and widespread natural enemies in agroecosystems [61]. All of these trends have prompted increasing interest in new management tools as well as avoidance of insecticide resistance in this important group.

The current study addressed the harlequin bug gene space from both quantitative and qualitative perspectives. That only one biological replicate’s transcriptome was sequenced implies that any inferred fold changes in gene expression per RNA-Seq data are at most suggestive of differential expression (DE), but these cannot support the notion of true DE with any statistical significance. However, a subset of transcripts exhibiting high fold changes was further probed using quantitative real-time PCR combined with rigorous statistical analyses—for these cases, DE could be asserted, and these findings represent compelling targets on which to perform RNAi-based reverse genetics. The lack of biological replication among RNA-Seq data per se has no impact on qualitative results presented herein; in particular, these qualitative findings afford useful insights into the inherent enzymatic capacity of the harlequin bug to detoxify xenobiotic compounds and to synthesize terpenoid compounds.

*M. histrionica* appears to possess four distinct Delta-class GST genes, although these resolve to two pairs, each of which contain two genes exhibiting very high levels of sequence similarity. Whether these correspond to recent duplication events or to potential RNA sequencing and assembly artifacts will require exploring the genome of *M. histrionica*, which is beyond the scope of this study. The *H. halys* genome seems to encode two Delta-type GSTs. These counts are lower than what has been seen in other hemipterans: *M. persicae* has eight and *Acyrthosiphon pisum* has ten such genes [62]. They are also lower than what has been observed in the Dipterans *Drosophila melanogaster* (11) and *Anopheles gambiae* (12) [63,64], although slightly higher than that seen in the Hymenopteran species, *Apis mellifera* (one; [23]). The low count of Delta-type GSTs in both harlequin bug and brown marmorated stink bug makes them appealing as knockdown/knockout targets for transcriptional disruption. Their potential utility may, however, be overshadowed by the generally highly conserved nature of genes from the Delta (as well as Epsilon) cytosolic GST subtypes, thereby incurring risks of off-target effects in practical applications. Transcriptome sequencing and analysis of closely related insect taxa should help clarify the extent to which such off-target gene silencing risks might manifest in unanticipated ecological damage.

Some *H. halys* scaffolds contain multiple β-esterase genes in close proximity, specifically scaffolds 261 and 2592, which comprise tandem arrays of eleven and six β-esterase genes, respectively. On both scaffolds 261 and 2592, *M. histrionica* β-esterase proteins mapped to three distinct, homologous β-esterase *H. halys* loci. This may suggest a potentially elevated β-esterase gene copy number in *H. halys* when compared to *M. histrionica*. However, fifteen unique *M. histrionica* carboxylesterase-associated protein sequences that did not map to the *H. halys* genome via GenomeThreader analysis are all located within the carboxylesterase clade (Supplementary Figure S3). These unmapped *M. histrionica* carboxylesterase-associated protein sequences may represent multiple *M. histrionica*-specific carboxylesterases. Currently, with only *M. histrionica* transcript data, it is not possible to identify novel gene duplication or to identify divergent homologs.

Inspection of genome sequence organization hints towards an increase in copy number of *H. halys* β-esterase genes through tandem gene duplication, which may have been effected by chromosomal slippage during DNA replication, for example. Results considered herein suggest that tandem duplication of β-esterase genes is also present in *M. histrionica*, albeit to a lesser extent than observed in *H. halys*. Whether such shared duplications occurred in a common ancestor or were realized as independent events subsequent to speciation remains uncertain, and will require genome sequence analysis of a broader taxonomic sampling of pentatomid species to resolve.

It is possible that β-esterase gene duplication is a common method of generating insecticide resistance in the Hemiptera. Gene duplication and over expression of β-esterases has been observed as one of the primary insecticide resistance mechanisms in *M. persicae* [65,66]. The presence of numerous carboxylesterase genes being actively transcribed in cultures of *H. halys* and *M. histrionica*, neither of which have been exposed to insecticides for at least 30 and 10 generations, respectively, suggests these constitute a standing defense against insecticide and/or other toxin exposure. Future investigation of differential expression between insecticide exposed and non-exposed groups could elucidate gene expression regulation associated with insecticide exposure. Identification of up regulated genes in response to insecticide exposure could help narrow the field of potential β-esterase gene targets, as well as identify regulation mechanisms that may also be of interest for combatting insecticide resistance.

Harlequin bug cytochrome P450s are most similar to brown marmorated stink bug (BMSB) P450s. BMSB has 128 named P450s in 26 families and 11 fragments that were too short to name. Harlequin bug has 87 named P450s and 17 fragments in 25 families. Sixty-three (72%) were considered orthologs. The differences occurred in gene clusters where there was not a 1:1 relationship between the species. The BMSB had more P450s in some large gene blooms like CYP6LV (20:9) and CYP6LT (8:4). One CYP3 clan family CYP3228A1 was found in BMSB but not in harlequin bug. Only two new subfamilies were present in harlequin bug CYP3226C and CYP4KC1. The harlequin bug data are based on transcriptome sequences. If some genes were not expressed they would not be represented. This may explain some of the differences. The large number of genes in the CYP3 (43 genes) and CYP4 (30 genes) clans provides the raw material for developing pesticide resistance. The genes in the CYP2 and mito clans might be good targets to block molting (Halloween genes and CYP18) and juvenile hormone synthesis (CYP15). Inhibition of the CYP4G P450s could act to block or reduce cuticular hydrocarbon synthesis, though this subfamily has six genes so inhibition might be difficult to achieve. Differential P450 expression in antennae could identify potential pheromone metabolizing P450s. Although the mating process in *M. histrionica* is not exclusively controlled by olfaction, but also via behavioral cues including visual and vibrational signals, inhibitors of P450s may nonetheless have utility in disrupting successful mating.

In the core MVA terpenoid biosynthetic pathway of *M. histrionica* and *H. halys*, all enzymes except for acetoacetyl-CoA synthase appear to be represented by single-copy genes, which is consistent with MVA pathways from other insects (e.g., [67,68,69]). On the contrary, enzymes involved in the conversion of FDP to farnesoic acid in JH biosynthesis are known to be encoded by families of genes with expression in different tissues based on the importance of farnesol and farnesal homeostasis for a variety of cellular functions [70]. Accordingly, we found isoforms of *M. histrionica* and *H. halys* farnesol and farnesal dehydrogenase; however, we were unable to identify orthologs of farnesyl diphosphatase. Furthermore, in agreement with findings from other insects [70], harlequin bug and brown marmorated stink bug methyl farnesoate epoxidase appears to be represented by a single-copy gene transcript; unexpectedly, we found several potential isoforms for juvenile hormone acid methyltransferase. To build a biosynthetic branch for the formation of murgantiol, it is possible that the pathway is initiated by one of the two identified FDP synthases because of the higher male specific expression of this gene (at least for *M. histrionica*). It can further be assumed that P450 enzymes with similarity to methyl farnesoate epoxidase are involved in the final epoxidation step in murgantiol biosynthesis because of the same position of the epoxy group at C10-C11 in both pheromone and JH molecules. Further comparative expression profiling paired with gene functional analysis will be required to elucidate the murgantiol biosynthetic pathway in *M. histrionica*.

## 5. Conclusions

This study generated the first high-volume collection of transcriptome sequence data for the important insect pest *Murgantia histrionica*. A number of gene targets for transcriptional disruption were identified, which will require further experimental study in an in vivo context. These genes include elements of three families implicated in the development of resistance to synthetic insecticides (glutathione S-transferases, carboxyesterases and cytochrome P450s), as well as genes involved pheromone biosynthesis pathways. These data will be of further utility in studies addressing additional gene families, in performing comparative transcriptomics among various insect species, and in supporting gene annotation efforts for any possible genome sequencing efforts made specifically for the harlequin bug.

## Figures and Tables

**Table 1:** Sequencing data volumes achieved, pre- and post-normalization. A paired-end sequencing strategy was used, with a targeted read length of 100 bp.

	Raw Sequence Data		Data Post-Normalization	
	*read pairs*	*bases*	*read pairs*	*bases*
2^nd^ Instar	213,817,714	42,763,542,800	13,268,461	2,653,692,200
4^th^ Instar	222,117,185	44,423,437,000	15,199,439	3,039,887,800
Female	205,127,388	41,025,477,600	11,786,560	2,357,312,000
Male	218,215,787	43,643,157,400	14,191,526	2,838,305,200
Totals	859,278,074	171,855,614,800	54,445,986	10,889,197,200

**Table 2:** The 25 most abundant Pfam families encountered in each of the gold-tier and complete harlequin bug PUT datasets.

Gold-Tier PUTs (Trimmed per Homology Info)			All PUT Sequences (Untrimmed)		
*Pfam hit*	*Pfam Description*	*Counts*	*Pfam hit*	*Pfam Description*	*Counts*
PF00069.23	Protein kinase domain	511	PF00078.25	Reverse transcriptase (RNA-dependent DNA polymerase)	3272
PF07714.15	Protein tyrosine kinase	504	PF00096.24	Zinc finger, C2H2 type	1424
PF13894.4	C2H2-type zinc finger	383	PF13894.4	C2H2-type zinc finger	1292
PF00096.24	Zinc finger, C2H2 type	381	PF13465.4	Zinc-finger double domain	1262
PF13857.4	Ankyrin repeats (many copies)	324	PF00069.23	Protein kinase domain	1256
PF13465.4	Zinc-finger double domain	323	PF12796.5	Ankyrin repeats (3 copies)	1245
PF12796.5	Ankyrin repeats (3 copies)	323	PF13637.4	Ankyrin repeats (many copies)	1209
PF00400.30	WD domain, G-beta repeat	323	PF13857.4	Ankyrin repeats (many copies)	1203
PF13637.4	Ankyrin repeats (many copies)	319	PF07714.15	Protein tyrosine kinase	1199
PF00023.28	Ankyrin repeat	316	PF07690.14	Major Facilitator Superfamily	1183
PF13606.4	Ankyrin repeat	313	PF00023.28	Ankyrin repeat	1171
PF07690.14	Major Facilitator Superfamily	288	PF13606.4	Ankyrin repeat	1154
PF12894.5	Anaphase-promoting complex subunit 4 WD40 domain	280	PF13909.4	C2H2-type zinc-finger domain	956
PF13927.4	Immunoglobulin domain	268	PF01359.16	Transposase (partial DDE domain)	844
PF14531.4	Kinase-like	266	PF00400.30	WD domain, G-beta repeat	834
PF13895.4	Immunoglobulin domain	261	PF13927.4	Immunoglobulin domain	741
PF00047.23	Immunoglobulin domain	259	PF00083.22	Sugar (and other) transporter	734
PF07679.14	Immunoglobulin I-set domain	257	PF07679.14	Immunoglobulin I-set domain	676
PF01926.21	50S ribosome-binding GTPase	256	PF00047.23	Immunoglobulin domain	636
PF07686.15	Immunoglobulin V-set domain	255	PF13895.4	Immunoglobulin domain	635
PF08477.11	Ras of Complex, Roc, domain of DAPkinase	244	PF00005.25	ABC transporter	620
PF00071.20	Ras family	238	PF00076.20	RNA recognition motif (a.k.a. RRM, RBD, or RNP domain)	611
PF00076.20	RNA recognition motif (a.k.a. RRM, RBD, or RNP domain)	235	PF00067.20	Cytochrome P450	590
PF12799.5	Leucine Rich repeats (2 copies)	234	PF00665.24	Integrase core domain	578
PF00025.19	ADP-ribosylation factor family	211	PF07686.15	Immunoglobulin V-set domain	546

**Table 3:** Gold-tier transcripts exhibiting the greatest gene expression fold changes within each of the three harlequin bug mRNA population comparisons performed. Expression levels were conveyed using the Transcripts Per Million (TPM) measure, and binary logarithms were used to rank sample-specific ratios of these amounts. A floor on TPM values of 5.0 was imposed.

**2nd Instar Expression Relative to 4th Instar**				
log2FC	Direction	2nd Instar	4th Instar	NR Gene
5.1284	up	253.24	7.24	adult-specific cuticular protein ACP-20-like
4.6366	up	1055.89	42.45	*uncharacterized protein LOC106688964*
3.6726	up	366.36	28.73	tubulin beta-1 chain
3.5047	up	380.01	33.48	protein takeout-like
3.3727	up	192.25	18.56	GTP cyclohydrolase 1 isoform X1
5.3563	down	8.17	334.68	heat shock 70 kDa protein cognate 4
5.3279	down	7.17	287.99	acyl-CoA Delta(11) desaturase-like isoform X1
5.2056	down	6.33	233.59	*uncharacterized protein LOC106679388*
4.7751	down	9.21	252.18	lysosomal alpha-mannosidase
4.6475	down	6.05	151.63	*uncharacterized protein LOC106683993*
**Female Expression Relative to Male**				
log2FC	Direction	Female	Male	NR Gene
4.1357	up	375.11	21.34	heat shock 70 kDa protein cognate 4
3.5908	up	104.22	8.65	JH acid O-methyltransferase-like isoform X1
2.8439	up	88.45	12.32	*uncharacterized protein LOC106679932*
2.7760	up	124.18	18.13	troponin C, isoform 1-like
2.7262	up	34.21	5.17	adult-specific cuticular protein ACP-20-like
3.9320	down	570.39	8706.01	putative odorant-binding protein 4
2.7080	down	5.58	36.46	E3 ubiquitin-protein ligase TRIM37-like isoform X2
2.2543	down	6.29	30.01	exocyst complex component 7
1.9412	down	6.51	25.00	DDB1- and CUL4-associated factor 6-like
1.9045	down	7.18	26.88	exosome complex component CSL4
**Adult Expression Relative to Nymph**				
log2FC	Direction	Adult	Nymph	NR Gene
3.8701	up	5288.23	361.66	putative odorant-binding protein 4
3.7040	up	105.04	8.06	inositol monophosphatase 2-like
3.4889	up	98.80	8.80	*uncharacterized protein LOC106686819*
3.4091	up	78.93	7.43	*uncharacterized protein LOC106691169*
3.0628	up	264.22	31.62	NADP-dependent malic enzyme-like isoform X3
6.0853	down	44.80	3041.89	*uncharacterized protein LOC106688633*
4.3486	down	5.41	110.22	pro-resilin-like
3.7376	down	13.83	184.48	probable antibacterial peptide
3.6876	down	9.00	115.96	cuticle protein 18.6, isoform B
3.2451	down	11.07	104.96	GTP cyclohydrolase 1 isoform X1

**Table 4:** *M. histrionica* PUTs encoding cytochrome P450 enzymes that appear to have sex-preferential expression patterns. Those among the most predominantly expressed in female adults are highlighted in yellow; those more predominantly expressed in males are highlighted in orange; and those having expression values of at least 5 TPM in both sex-specific samples, as well as at least a two-fold expression differential, are presented with grey highlighting.

♂ (TPM)	♀ (TPM)	|log_2_(♂:♀)|	Direction	HBUG PUT Id	Best NR hit
0.03	18.87	9.2969	-	472510	XP_014270541.1 (CYP4GZ4)
0.07	24.35	8.4424	-	473495	XP_014286441.1 (CYP6LT5)
0.40	72.44	7.5006	-	419699	XP_014273208.1 (CYP6LT7)
0.06	8.73	7.1849	-	103772	XP_014286439.1 (CYP6LT3)
0.27	19.68	6.1876	-	103769	XP_014286439.1 (CYP6LT3)
6.35	0.00	undefined	+	478727	XP_014293876.1 (CYP307B1)
4.71	0.00	undefined	+	173857	XP_014285590.1 (CYP3226B1)
9.95	0.01	9.9586	+	298833	XP_014293876.1 (CYP307B1)
4.30	0.07	5.9408	+	137898	XP_014288222.1 (CYP3227B4)
3.26	0.15	4.4418	+	521147	XP_014276563.1 (CYP3225B3)
5.85	0.61	3.2616	+	428429	XP_014279285.1 (CYP302A1)
38.09	6.24	2.6098	+	103771	XP_014286439.1 (CYP6LT3)
21.99	6.92	1.6680	+	504543	XP_014284935.1 (CYP4HB7)
15.69	5.20	1.5933	+	456532	XP_014285589.1 (CYP3226B1)
25.99	8.88	1.5493	+	486339	XP_014285590.1 (CYP3226B1)
14.45	5.10	1.5025	+	316472	XP_014274999.1 (CYP3224A2)
15.06	5.51	1.4506	+	165088	XP_014271425.1 (CYP6LU1)
13.48	6.26	1.1066	+	212119	XP_014284933.1 (CYP4HB6)
7.51	15.33	1.0295	-	504542	XP_014284935.1 (CYP4HB7)

**Table 5:** *M. histrionica* PUTs encoding terpene biosynthesis-related genes (no genes for ubiquinone or dolichol biosynthesis were considered).

Enzyme Name	Query Sequences (multiple taxa)	Transcript Identified (*M. histrionica*)	Blastx Support (*H. halys*)
Acetoacetyl-CoA thiolase	XM_014419845,XM_014386017,XM_015512081,AK403218	HBug_USDA-ARS_IIBBL.267134	XP_014294739.1
		HBug_USDA-ARS_IIBBL.43170	XP_014277769.1
HMG-CoA synthase	XM_014416338,X70034,AB733009	HBug_USDA-ARS_IIBBL.375421	XP_014277503.1
HMG-CoA reductase	X70034,XM_014424783,XM_014391838,XM_015521221	HBug_USDA-ARS_IIBBL.421664	XP_014280269.1
Mevalonate kinase	XM_014416757,XM_014391202,GEDC01029638,XM_012431690	HBug_USDA-ARS_IIBBL.227208	XP_014272243.1
Phosphomevalonate kinase	XM_014416475,XM_014398812,GECZ01001991,GEBQ01010256	HBug_USDA-ARS_IIBBL.270718	XP_014271961.1
Diphosphomevalonate decarboxylase	XM_018479978,XM_014434730,XM_014399537,GEBQ01002905	HBug_USDA-ARS_IIBBL.428020	XP_014290216.1
IDP Isomerase	XM_014415973,XP_014247428,GECU01023093,AK417896	HBug_USDA-ARS_IIBBL.92242	XP_014271459.1
FDP Synthase	XP_014289225	HBug_USDA-ARS_IIBBL.420512	XP_014276183.1
		HBug_USDA-ARS_IIBBL.414919	XP_014276401.1
Farnesyl diphosphatase	NP_572760.1	*No homologs detected*	
Farnesol dehydrogenase	XP_014292348	HBug_USDA-ARS_IIBBL.414590	XP_014286519.1
		HBug_USDA-ARS_IIBBL.376500	XP_014286525.1
		HBug_USDA-ARS_IIBBL.328207	XP_014286524.1
Farnesal dehydrogenase	KC243495	HBug_USDA-ARS_IIBBL.79640	XP_014292700.1
		HBug_USDA-ARS_IIBBL.14716	XP_014272618.1
Juvenile hormone acid methyltransferase	XP_014293044,XP_001651876	HBug_USDA-ARS_IIBBL.485486	XP_014290953.1
		HBug_USDA-ARS_IIBBL.519494	XP_014293044.1
		HBug_USDA-ARS_IIBBL.346622	XP_014283772.1
Methyl farnesoate epoxidase	XP_014283057	HBug_USDA-ARS_IIBBL.517163	XP_014283057.1

**Table 6:** Gene expression levels for *Murgantia histrionica* and *Halyomorpha halys* genes associated with terpene biosynthesis. RSEM-calculated expression values are conveyed in units of Transcripts per Million (TPM), and comparisons correspond to binary logs of TPM ratios.

Harlequin Bug											
Annotation	2nd	4th	4th:2nd	♂	♀	♀ : ♂	Nymphs	Adults	Adults:Nymphs	Overall	Transcript ID
Acetoacetyl-CoA thiolase	176.92	131.53	-0.43	211.58	344.29	0.70	154.39	267.85	0.79	211.99	HBug_USDA-ARS_IIBBL.267134
Acetoacetyl-CoA thiolase	41.01	28.64	-0.52	21.84	33.13	0.60	34.61	26.66	-0.38	30.56	HBug_USDA-ARS_IIBBL.43170
HMG-CoA reductase	15.83	12.42	-0.35	29.97	22.95	-0.39	14.10	27.05	0.94	20.60	HBug_USDA-ARS_IIBBL.421664
HMG-CoA synthase	0.53	0.60	0.18	0.27	0.45	0.74	0.57	0.35	-0.70	0.45	HBug_USDA-ARS_IIBBL.375421
Mevalonate kinase	0.11	0.28	1.35	0.05	0.33	2.72	0.22	0.13	-0.76	0.19	HBug_USDA-ARS_IIBBL.227208
Phosphomevalonate kinase	4.51	2.76	-0.71	6.08	3.08	-0.98	3.59	4.76	0.41	4.15	HBug_USDA-ARS_IIBBL.270718
Diphosphomevalonate decarboxylase	0.05	0.12	1.26	0.13	0.11	-0.24	0.09	0.14	0.64	0.15	HBug_USDA-ARS_IIBBL.428020
IDP Isomerase	20.22	10.48	-0.95	15.04	13.73	-0.13	15.32	14.53	-0.08	14.92	HBug_USDA-ARS_IIBBL.92242
FDP Synthase	3.44	0.41	-3.07	46.44	3.51	-3.73	1.92	28.48	3.89	15.29	HBug_USDA-ARS_IIBBL.420512
FDP Synthase	25.04	23.37	-0.10	32.47	31.56	-0.04	23.79	32.17	0.44	27.98	HBug_USDA-ARS_IIBBL.414919
Farnesol dehydrogenase	0.16	0.15	-0.09	0.00	0.00	undefined	0.16	0.00	- ∞	0.08	HBug_USDA-ARS_IIBBL.414590
Farnesol dehydrogenase	0.00	0.13	+ ∞	0.07	0.45	2.68	0.03	0.15	2.32	0.09	HBug_USDA-ARS_IIBBL.328207
Farnesol dehydrogenase	13.27	9.88	-0.43	7.78	7.54	-0.05	11.57	7.81	-0.57	9.65	HBug_USDA-ARS_IIBBL.376500
Farnesal dehydrogenase	0.29	0.20	-0.54	8.14	12.84	0.66	0.24	10.14	5.40	5.20	HBug_USDA-ARS_IIBBL.14716
Farnesal dehydrogenase	3.29	9.14	1.47	4.99	5.77	0.21	6.24	5.30	-0.24	5.78	HBug_USDA-ARS_IIBBL.79640
Juvenile hormone acid methyltransferase	0.04	0.00	- ∞	0.12	0.03	-2.00	0.00	0.09	+ ∞	0.00	HBug_USDA-ARS_IIBBL.519494
Juvenile hormone acid methyltransferase	3.04	10.78	1.83	4.97	64.51	3.70	6.87	30.65	2.16	17.85	HBug_USDA-ARS_IIBBL.485486
Juvenile hormone acid methyltransferase	0.03	1.22	5.35	0.09	0.28	1.64	0.68	0.17	-2.00	0.45	HBug_USDA-ARS_IIBBL.346622
Methyl farnesoate epoxidase	0.39	0.12	-1.70	0.18	0.14	-0.36	0.25	0.17	-0.56	0.21	HBug_USDA-ARS_IIBBL.517163
Brown Marmorated Stink Bug											
Annotation	2nd	4th	4th:2nd	♂	♀	♀ : ♂	Nymphs	Adults	Adults:Nymphs	Overall	Protein / RNA IDs
Acetoacetyl-CoA thiolase	175.06	168.71	-0.05	793.94	942.05	0.25	171.71	864.84	2.33	452.10	XP_014294739.1 / XM_014439253.1
Acetoacetyl-CoA thiolase	70.48	65.24	-0.11	147.12	185.97	0.34	67.72	165.71	1.29	107.36	XP_014275331.1 / XM_014419845.1
HMG-CoA reductase	18.12	21.68	0.26	44.27	25.96	-0.77	20.00	35.52	0.83	26.28	XP_014280269.1 / XM_014424783.1
HMG-CoA synthase	0.00	0.00	undefined	0.44	1.40	1.67	0.00	1.28	+ ∞	0.54	XP_014277503.1 / XM_014422017.1
Mevalonate kinase	8.00	8.54	0.09	12.13	7.82	-0.63	8.29	10.07	0.28	9.01	XP_014272243.1 / XM_014416757.1
Phosphomevalonate kinase	5.97	4.46	-0.42	7.02	4.80	-0.55	5.17	5.96	0.21	5.49	XP_014271961.1 / XM_014416475.1
Diphosphomevalonate decarboxylase	3.96	3.44	-0.20	4.90	3.98	-0.30	3.72	4.46	0.26	4.04	XP_014290216.1 / XM_014434740.1
IDP Isomerase	13.51	10.56	-0.36	11.89	14.15	0.25	11.96	12.97	0.12	12.37	XP_014271459.1 / XM_014415973.1
FDP Synthase	3.62	8.08	1.16	15.70	18.66	0.25	5.97	17.12	1.52	10.48	XP_014276183.1 / XM_014420697.1
FDP Synthase	12.52	14.96	0.26	13.13	15.78	0.27	13.80	14.40	0.06	14.04	XP_014276401.1 / XM_014420915.1
FDP synthase	0.04	0.16	2.00	0.80	0.04	-4.32	0.10	0.44	2.14	0.24	XP_014289203.1 / XM_014433717.1
FDP synthase	0.29	0.29	0.00	0.35	0.22	-0.67	0.29	0.28	-0.05	0.29	XP_014289225.1 / XM_014433739.1
Farnesol dehydrogenase	204.63	77.01	-1.41	35.70	46.26	0.37	137.30	40.68	-1.75	98.17	XP_014286519.1 / XM_014431033.1
Farnesol dehydrogenase	78.56	47.27	-0.73	43.79	43.94	0.00	62.05	43.86	-0.50	54.69	XP_014286524.1 / XM_014431038.1
Farnesol dehydrogenase	107.93	53.10	-1.02	44.15	27.32	-0.69	79.01	36.10	-1.13	61.65	XP_014286525.1 / XM_014431039.1
Farnesal dehydrogenase	28.66	94.12	1.72	35.72	49.72	0.48	63.19	42.42	-0.57	54.79	XP_014272618.1 / XM_014417132.1
Farnesal dehydrogenase	49.42	54.55	0.14	57.72	65.03	0.17	52.13	61.22	0.23	55.81	XP_014292700.1 / XM_014437214.1
Juvenile hormone acid methyltransferase	0.13	0.06	-1.12	0.22	34.99	7.31	0.09	16.85	7.55	6.87	XP_014293044.1 / XM_014437558.1
Juvenile hormone acid methyltransferase	46.90	45.31	-0.05	22.24	79.28	1.83	46.06	49.52	0.10	47.46	XP_014290953.1 / XM_014435467.1
Juvenile hormone acid methyltransferase	0.38	0.34	-0.16	0.79	0.18	-2.13	0.36	0.50	0.47	0.41	XP_014283772.1 / XM_014428286.1
Methyl farnesoate epoxidase	1.15	0.59	-0.96	0.16	0.26	0.70	0.86	0.21	-2.03	0.59	XP_014283057.1 / XM_014427571.1

## Supplementary Materials

The following are available online at http://www.mdpi.com/2075-4450/8/2/55/s1, Supplementary Figure S1 (GO term abundances (full ontology)), Figure S2 (GO-Slim term abundances) and Figure S3 (Maximum likelihood based carboxylesterase phylogeny for *Halyomorpha halys* and *Murgantia histrionica* proteins); and Table S1: Gene expression levels of putative unique transcripts exhibiting at least two-fold expression differentials among three comparisons.
