# Supplementary material for: A Transcriptome Survey Spanning Life Stages and Sexes of the Harlequin Bug, Murgantia histrionica
Source: Insects. 2017 May 25;8(2):55. doi: 10.3390/insects8020055 (PMC5492069; doi:10.3390/insects8020055)
Supplement: Supplementary file 1 [file insects-08-00055-s001.zip › insects-189686-supplementary-Figures.docx]

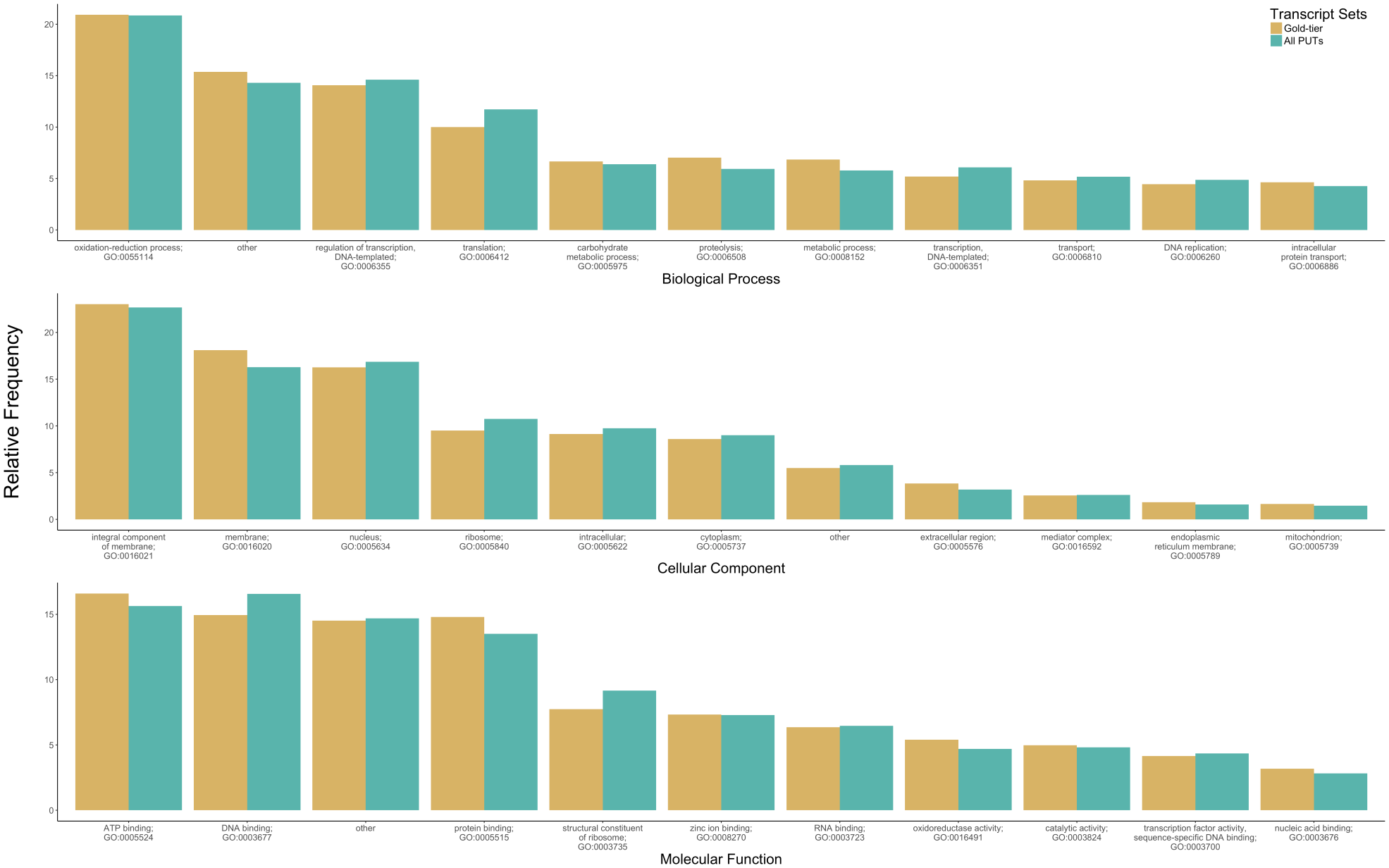


**Supplementary Figure S1.** GO term abundances (full ontology). Bar charts present the relative abundance of the ten most frequently encountered gene ontology (GO) terms for both the 19,303 gold-tier PUT subset, as well as the complete 523,103 PUT set. GO terms contribute additional annotation information for inferred harlequin bug genes, and were stratified along the ontology’s three primary aspects: biological process, cellular component and molecular function.


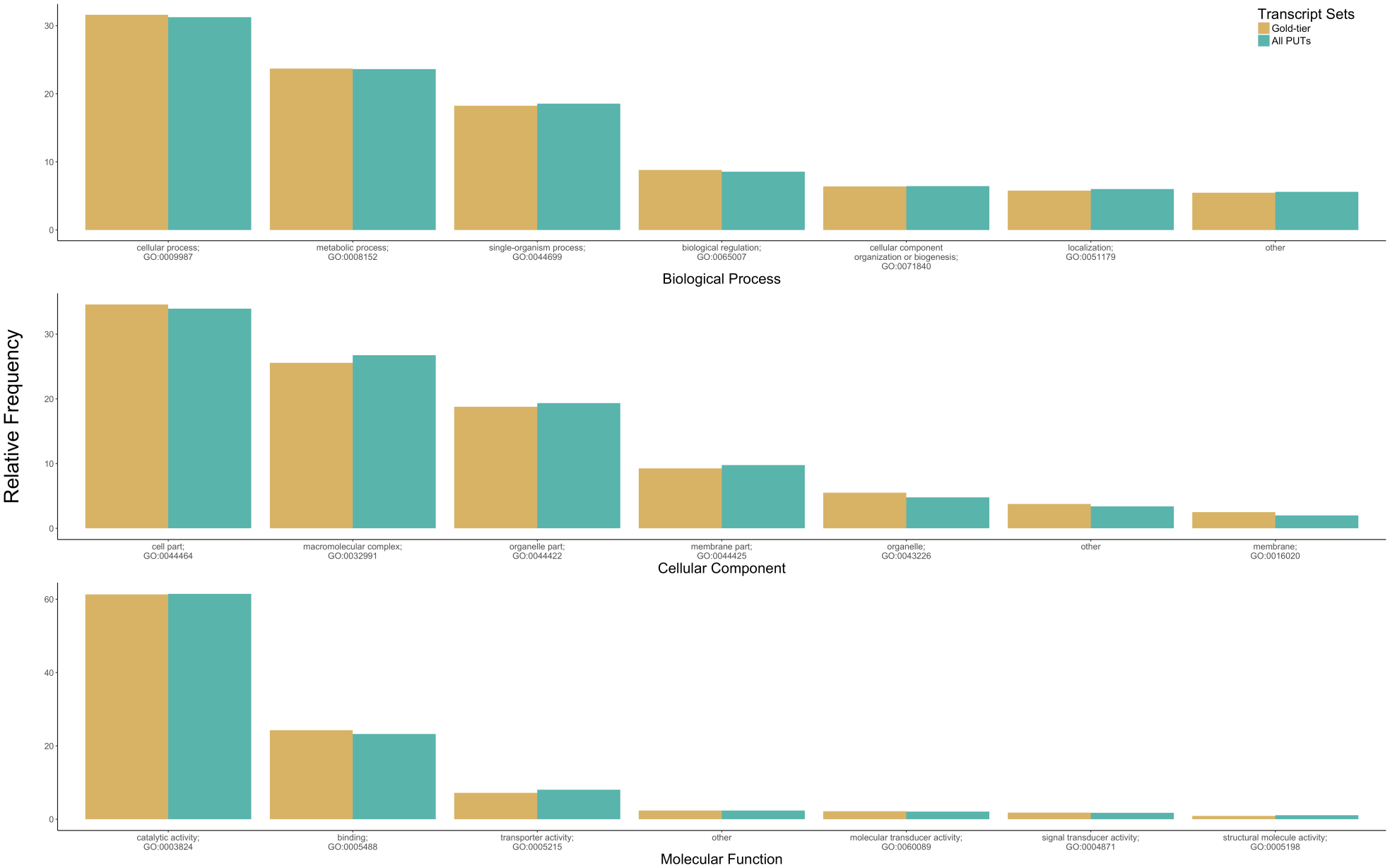
**Supplementary Figure S2.** GO-Slim term abundances. Shown are bar charts presenting the relative abundance of the six most frequently encountered GO-Slim terms for both the 19,303 gold-tier PUT subset, as well as the complete 523,103 PUT set. GO-Slim terms offer a higher-level, less-granular perspective on the functional capacity contained in the harlequin bug gene set relative to terms derived from the full gene ontology, and they correspond to penultimate ancestral nodes retrieved from the ontology’s directed acyclic graph representation. These slimmed terms are stratified with respect to the ontology’s three primary domains: biological process, cellular component and molecular function.


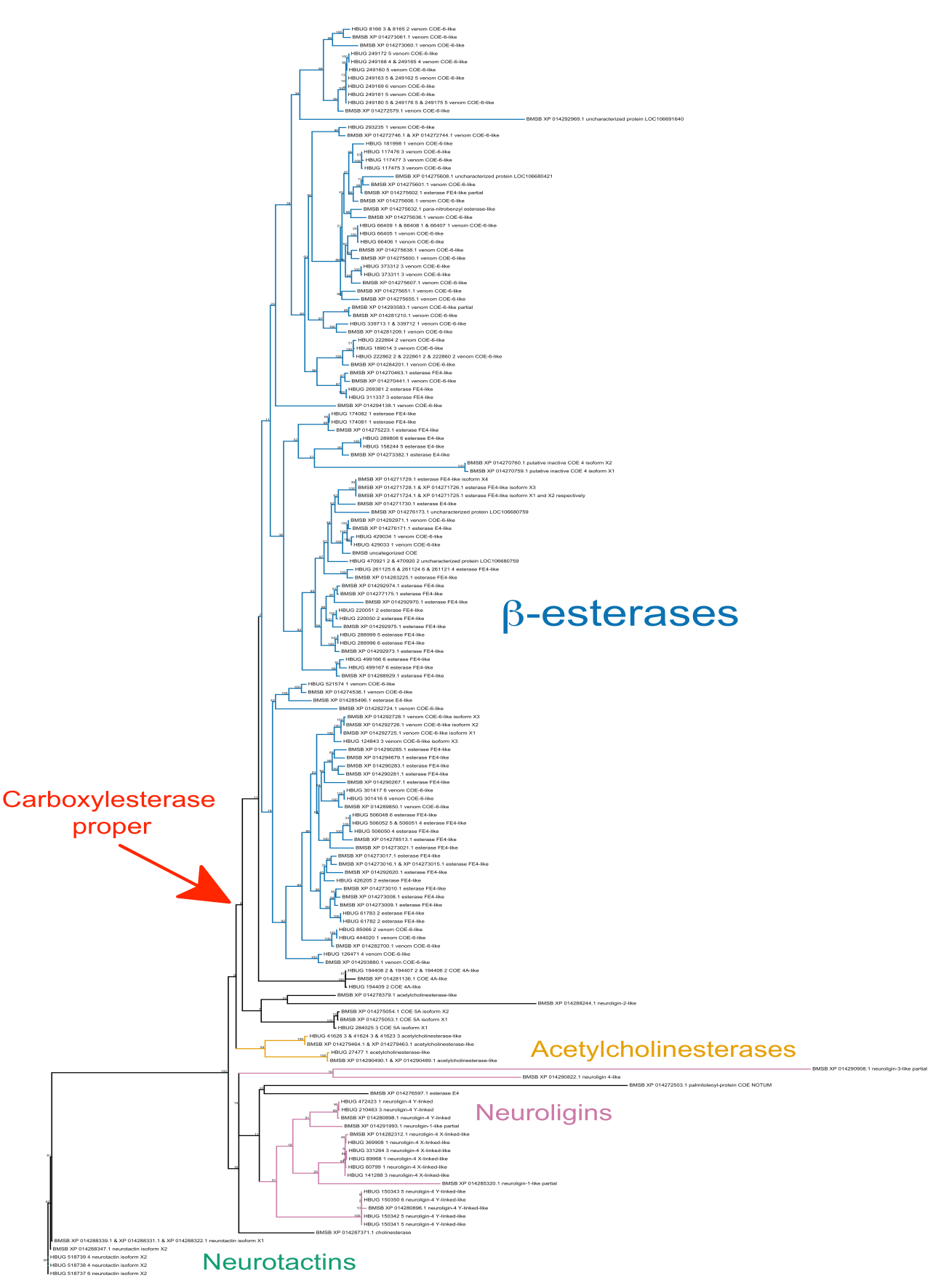


**Supplementary Figure S3.** Maximum likelihood-based carboxylesterase phylogeny for *Halyomorpha halys* and *Murgantia histrionica* proteins. Neurotactin (green), neuroligin (pink) and acetylcholinesterase (brown) sequences are all basal to the large carboxylesterase proper clade (red arrow) that contains 4A- and 5A-like carboxylesterases, as well as the large β-esterase subclade (blue). Bootstrap support (100 replicates) is indicated on branches.
